# Preclinical characterization of the efficacy and safety of biologic N-001 as a novel pain analgesic for post-operative acute pain treatment

**DOI:** 10.1038/s41598-023-38618-4

**Published:** 2023-07-21

**Authors:** Derek Allen, Samerender Nagam Hanumantharao, Rylie McDonell, Karen-Amanda Irvine, Peyman Sahbaie, David Clark, Paul Blum

**Affiliations:** 1 Neurocarrus Inc, Monterey, CA USA; 2 Microbiology and Environmental Toxicology, University of California-Santa Cruz, Santa Cruz, CA USA; 3 School of Biological Sciences, University of Nebraska-Lincoln, Lincoln, NE USA; 4 Stanford University School of Medicine, Stanford, CA USA; 5 VA Palo Alto Health Care, Palo Alto, CA USA

**Keywords:** Drug development, Preclinical research

## Abstract

Inhibition of actin remodeling in nerves modulates action potential propagation and therefore could be used to treat acute pain. N-001 is a novel protein analgesic engineered from several *C. Botulinum* toxins. N-001 targets sensory neurons through ganglioside GT1b binding and ADP-ribosylates G-actin reducing actin remodeling. The activity and efficacy of N-001 was evaluated previously in vitro and in a mouse inflammatory pain model. To assess the relevance of N-001 for treatment of acute post-surgical pain, the current study evaluated the efficacy of N-001 in a mouse hind-paw incision model by peri-incisional and popliteal nerve block administration combined with mechanical testing. N-001 provided relief of pain-like behavior over 3 days and 2 days longer than the conventional long-acting anesthetic bupivacaine. Preclinical safety studies of N-001 indicated the drug produced no toxic or adverse immunological reactions over multiple doses in mice. These results combined with past targeting results encourage further investigation of N-001 as an analgesic for post-operative pain management with the potential to function as a differential nociceptor-specific nerve block.

## Introduction

Post-operative pain is defined as the pain present in a patient after surgery^1^. Poorly managed postoperative pain can lead to lengthened hospital stay, complications and poor rehabilitation^2^. This can lead to chronic pain if the acute pain is prolonged and can reduce the quality of life. The use of opioid analgesia remains the mainstay in post-operative pain management^3^ and has led to an overuse of opioids to manage pain especially during chronic illness with a significant impact on clinical outcomes^4,5^. The opioid epidemic is one of the largest public health crises faced by the United States^6^. There are over 125 deaths per day resulting from drug overdose putting a socio-economic strain on the healthcare system^7–10^. The global postoperative market is rapidly expanding due to the increase in number of surgical procedures, severity of post-operative pain and adoption of pain management in emerging countries^11^. Hence, it is vital to explore other non-opioid treatment options to manage pain^12,13^.

Protein based therapeutics are viable alternatives to conventional opioids and may help address the opioid crisis^14,15^. Several peptides are under study that target ion channels, neuronal nicotinic receptors (nAChR), transient receptor potential channels (TRP), and different non-opioid G-protein coupled receptors (GPCRs)^16–19^. The overriding challenge with these drugs is their lack of specificity leading to off target effect combined with a limited effect due to rapid clearance^20,21^. A key component of inflammatory pain is cytoskeletal actin (F-actin) within sensory neurons which aids in structural and functional plasticity^22,23^. Reducing sensory neuron F-actin content by shifting actin treadmilling could block pain signaling by limiting ion channel activity. We have previously demonstrated the design and use of a novel recombinant protein (N-001) as a candidate inflammatory pain analgesic in vitro and in vivo^24,25^. N-001 is a recombinant protein engineered from several *Clostridium botulinum* toxins. The protein is composed of re-engineered C2II-C1 from the C2 toxin with a substituted C-terminal domain from the C1 toxin which is responsible for the binding and translocation of the active component called C2I^25^. C2I is an ADP ribosylase that post-translationally modifies G-actin ^26,27^. N-001 is specifically targeted to sensory neurons as demonstrated in vitro by selective uptake by sensory neurons and not motor neurons^24,25^. Additionally, the in vivo efficacy of C2C has been found to function against nociceptor-associated acute pain without an effect on motor function^24,25^. In addition, its large size and its local administration reduce diffusion and preclude central nervous system engagement^24,25^. These features make it an attractive prospective candidate for use as a post-operative analgesic.

It has previously been demonstrated that N-001 inhibits processes required for neural signaling in vitro^24^. Anesthetics block ion channel functions to inhibit the production of an action potential required for pain signaling^28^. N-001 has been shown to inhibit the influx of calcium influx through the depolymerization of actin^24^ and thus exhibits an anesthetic-like action. While local anesthetics are effective nociceptive pain control agents, they produce serious side effects including loss of motor function, numbness, and hypotension^29^. A differential nociception-specific nerve blockade would remedy this problem. A lidocaine derivative with a decreased ability for neuron entrance, QX-314, was combined with agonists for the nociceptor specific channel TRPV^30^. The TRPV agonist was specifically bound to TRPV channels to promote selective entry of the derivative into nociceptors^30^. While this combination had selective efficacy against nociceptors^30,31,44^, neurotoxicity and limited duration limited its utility^44^. In studies reported here, the in vivo nociceptor specificity and efficacy of N-001^24,25^ prompted attempts to evaluate its utility as a differential nociception-specific nerve block agent.

## Results

### Efficacy studies

#### Hind paw incision model

Our prior studies demonstrated N-001 relieved pain-like behaviors in the mouse formalin model, suggesting N-001 might exhibit efficacy towards other forms of pain. To determine the relevance of N-001 treatment for postoperative pain management, the drug was tested in a mouse hind-paw incision model by peri-incisional and popliteal nerve block administration combined with mechanical testing.

##### Peri-incisional intra plantar injection

**Figure 1:**
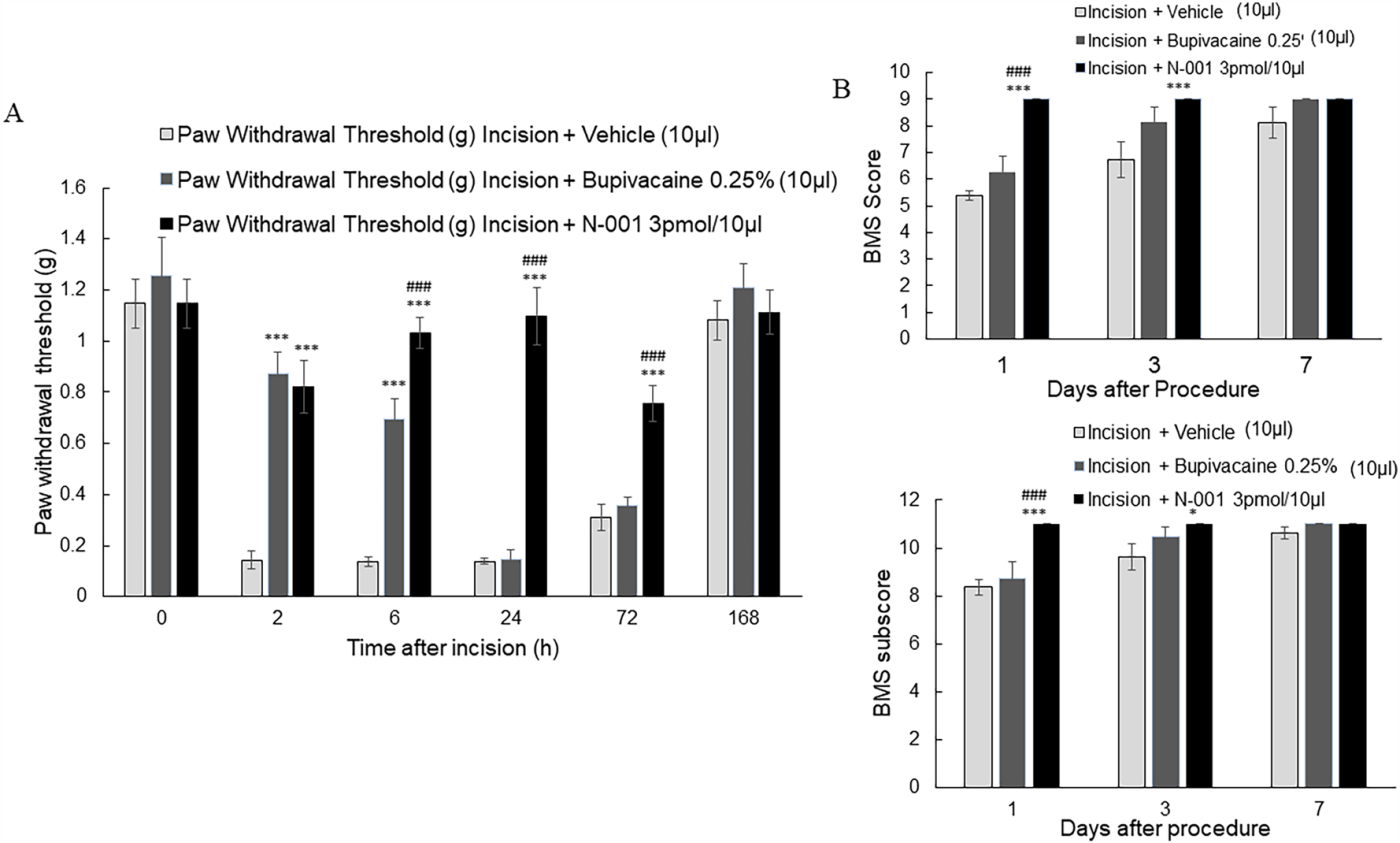
(**A**) Hind paw withdrawal threshold of C57Bl/6 J male mice after incision and vehicle/drug treatment. (**B**) Basso Motor Scale (BMS) and BMS Subscore of C57Bl/6 J male mice after incision and vehicle/drug treatment. Data represented as mean ± S.E.M and analyzed by repeated measures two-way ANOVA followed by Sidak post-hoc test (*** represents *p *< 0.001 ** represent *p *< 0.01, * represents *p *< 0.05 in all cases vs vehicle treatment. # represents *p *< 0.05 and ### represents *p *< 0.001 vs bupivacaine treatment, n = 8.).

N-001 reduced allodynia and improved gait in C57Bl/6 J male mice (Fig. 1A). Significant relief of pain-like behavior as measured by the Von Frey test was observed for N-001 and bupivacaine after 2 and 6 h. However, efficacy continued with N-001 for a significantly longer period than bupivacaine at both 24 and 72 h. Neither drug had an effect after 168 h. A similar pattern was observed regarding gait where N-001 exhibited significantly more benefit than bupivacaine after both 1 day and 3 days relative to carrier only (Fig. 1B).

**Figure 2:**
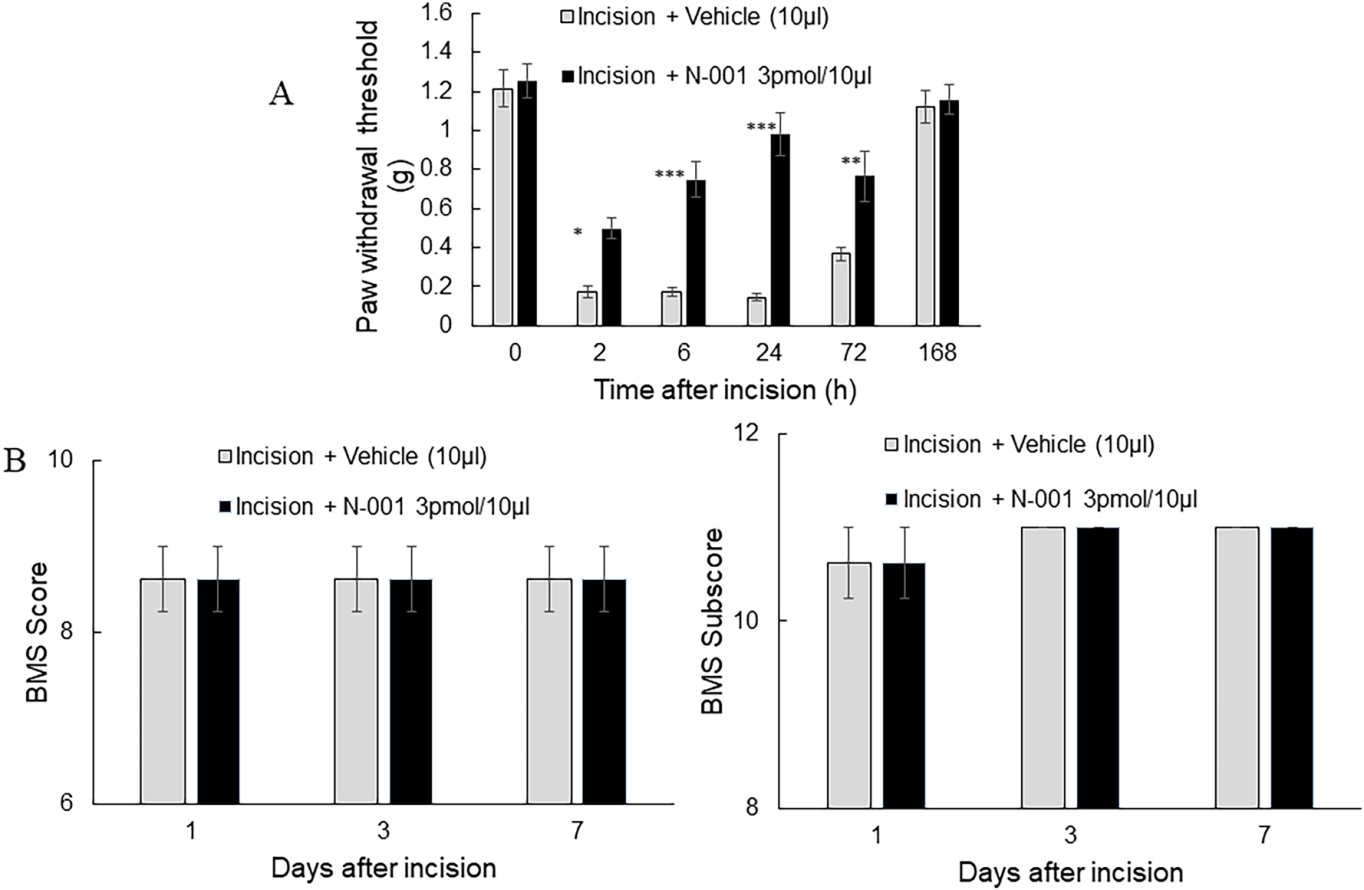
(**A**) Hind paw withdrawal threshold of C57Bl/6 J female mice after incision and vehicle/drug treatment. (**B**) Basso Motor Scale (BMS) of C57Bl/6 J female mice after incision and vehicle/drug treatment. Data represented as mean ± S.E.M and analyzed by repeated measures two-way ANOVA followed by Sidak post-hoc test (*** represents *p *< 0.001, ** represent *p *< 0.01, * represents *p *< 0.05 in all cases vs vehicle treatment, # represents *p *< 0.05, n = 8.).

A sham procedure control was performed using unincised C57Bl/6 J mice that showed no difference in Von Frey or gait test results (Supplementary fig. S1) between 2 and 168 h after surgery. A similar hind paw incision pain model was utilized on female mice to detect any difference in response based on sex (Fig. 2A). N-001 and a vehicle negative control were applied immediately along the suture line after the surgery was completed. Significant relief of pain-like behavior as measured by the Von Frey test was observed for N-001 after 2 h and continued until 72 h while the drug became ineffective at 168 h. A similar pattern was observed on gait where N-001 was active until 72 h (Fig. 2B). Compared to male mice, the female response to N-001 provided lower paw withdrawal threshold on the Von Frey at 2 h and 6-h. However, there were no overall statistically significant differences with efficacy between the sexes when analyzed with repeated measures two-way ANOVA for local treatment (Supplementary fig. S2). This response to pain-like behavior is consistent with the sex differences in pain perception where female mice are more sensitive to pain than male mice^32,33^.

#### Popliteal nerve block model

**Figure 3:**
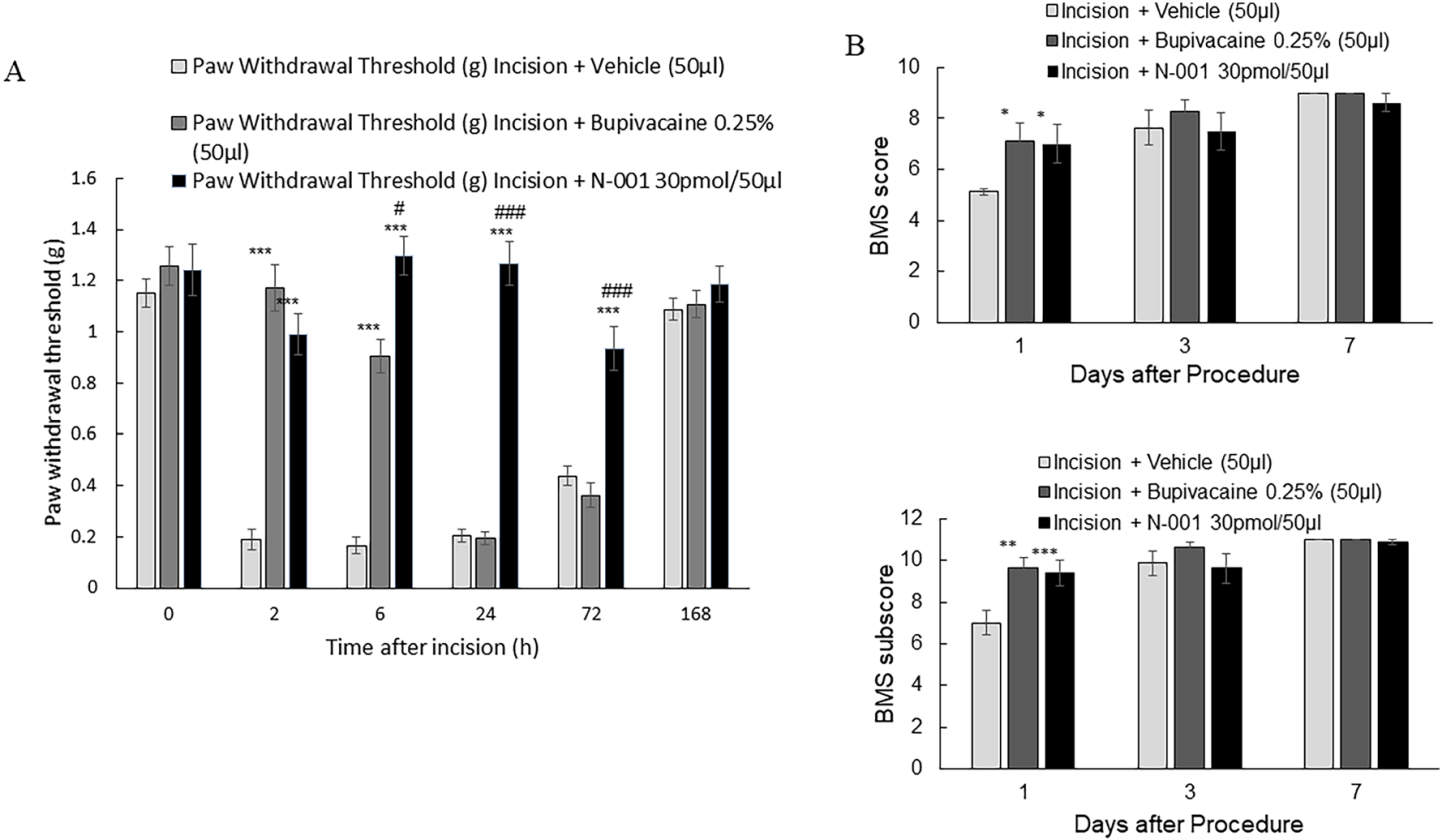
(**A**) Hind paw withdrawal threshold of C57BI/6 J male mice after incision procedure and vehicle/drug (50 μl Popliteal Block) treatment, n = 8. (**B**) Basso Motor Scale (BMS) of C57BI/6 J male mice after incision procedure and vehicle/drug (50 μl Popliteal Block) treatment, n = 8. Data represented as mean ± S.E.M and analyzed by repeated measures two-way ANOVA followed by Sidak post-hoc test (*** represents *p *< 0.001, ** represent *p *< 0.01, * represents *p *< 0.05 in all cases vs vehicle treatment, # represents *p *< 0.05 and ### represents *p *< 0.001 vs bupivacaine treatment, n = 8.).

Since N-001 was effective by peri-incisional administration, the utility of N-001 at a site distal from the surgical site was assessed by popliteal nerve block^34^ in male and female mice (n = 8). The tested nerve block compounds were administered in the popliteal space (located at the posterior surface of the knee) of the sciatic nerve, followed by an incision of the hind paw to initiate allodynia in the animals. A gait analysis study was included to quantify the differences in BMS score with the differences in application of N-001, either preoperatively before or after nerve block (Supplementary figure S3). N-001 reduced allodynia and improved gait in C57Bl/6 J male mice (Fig. 3A). Significant relief from pain-like behavior as measured by the Von Frey test was observed for N-001 and bupivacaine at 6 h and stopped for bupivacaine by 24-h. In contrast relief from pain-like behavior continued for 72-h for mice injected with N-001. Both drugs had no effect by 168 h. The gait test procedure resulted in significant positive differences for N-001 after 1 day compared to the negative controls while there was inconsistent gait recovery within the treatment groups of N-001 and bupivacaine (Fig. 3B).

**Figure 4:**
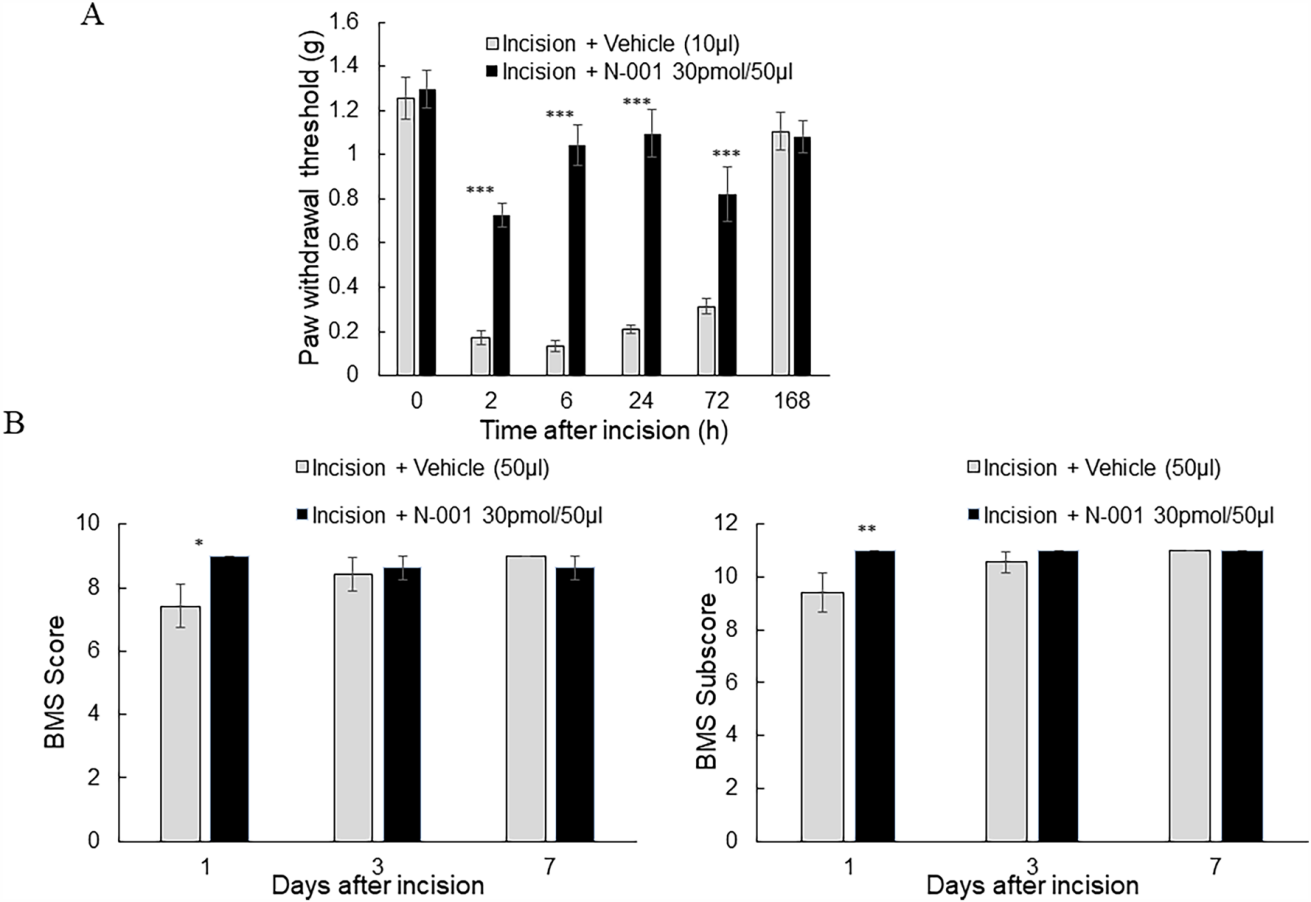
(**A**). Hind paw withdrawal threshold of C57BI/6 J female mice after incision procedure and vehicle/drug (50 μl Popliteal Block) treatment, n = 7–8. (**B**) Basso Motor Scale (BMS) of C57BI/6 J female mice after incision procedure and vehicle/drug (50 μl Popliteal Block) treatment, n = 7–8. Data represented as mean ± S.E.M and analyzed by repeated measures two-way ANOVA followed by Sidak post-hoc test (*** represents *p *< 0.001, ** represent *p *< 0.01, * represents *p *< 0.05 in all cases vs vehicle treatment).

A similar model was utilized with female mice to detect potential sex-dependent differences (Fig. 4A). N-001 and a vehicle negative control were applied through a popliteal nerve block prior to incision. Significant relief from pain-like behavior as measured by the Von Frey test was observed for N-001 after 2 h and then continued to 72 h. The drug became ineffective at 168 h. The gait test procedure resulted in significant positive differences only after 1 day compared to negative controls, with inconsistent gait relief within the treatment groups of N-001 or bupivacaine (Fig. 4B). There was an overall difference between males and females with how the mice perceived pain in their responses to nerve block, but a Sidak post hoc test did not show differences in hind paw withdrawal threshold and efficacy of N-001 (Supplementary fig. S4).

#### Safety studies

**Figure 5:**
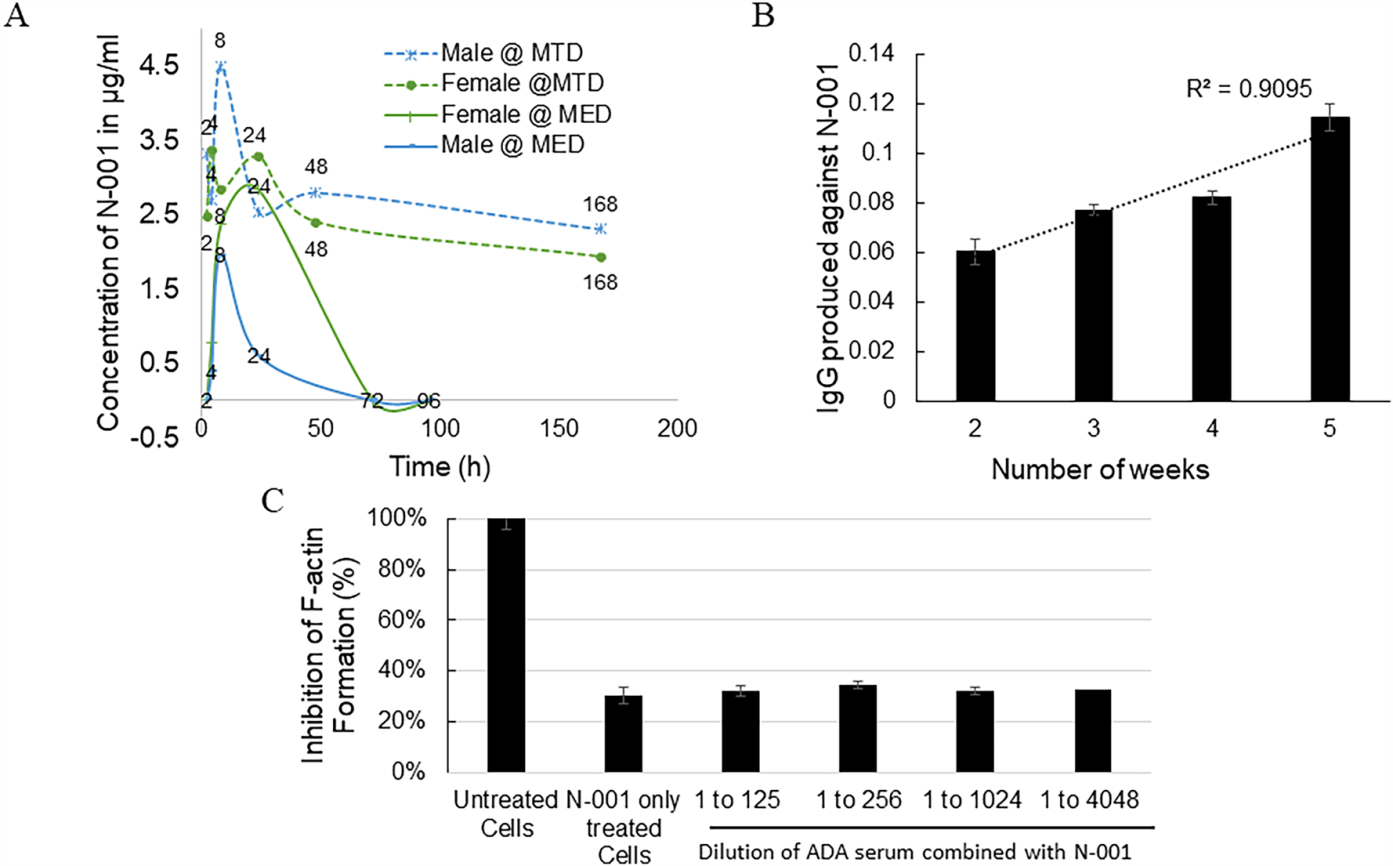
(**A**) The pharmacokinetics of N-001 was determined through IP injections in both male and female mice at different doses (MED and MTD) at 2,4,8,24,72 and 96 h. (**B**) The formation of antidrug antibodies (ADAs) was measured by ELISA. The mice were injected with N-001 at the times indicated. (**C**) A phalloidin potency assay was conducted for determining potential neutralization of N-001 by ADAs using SH-SY5Y cells. N-001 (2.4 pmol) was incubated with cells with a dilution series of ADA containing sera and compared to N-001 treated and untreated cells. The data are presented as means ± standard errors of the means (SEM) (error bars) from each titration performed in technical triplicates.

Whole blood and serum samples were used to check for toxicity in male and female mice (n = 4). The samples were analyzed for standard blood chemistry and cytology (42 parameters). The mice had no abnormal levels for the parameters checked. The mice additionally had no signs of distress or behavioral changes during the study (Supplementary data). The pharmacokinetics (PK) of the drug was analyzed to determine its pharmacological parameters. The lower limit of quantification of the assay to determine drug in blood serum was 50 ng/mL. The PK curves followed a non-linear clearance with a C_max_ at 24-h in females and 8-h in male mice for the mice injected at both MTD and MED (Fig. 5A). The current analytical method targeted the active part of the drug (C2I) using a primary antibody that was specific for the C2I region of N-001.

The adaptive immune system is activated generally 9–14 days in mice after immunization. Therefore, IgG antibody production was investigated after 4 successive booster doses (0.8 pmol (MED)/g body weight) in female mice (n = 12) to identify the presence of anti-drug antibodies (ADAs). There was a linear increase in the production of ADAs with increasing doses (Fig. 5B). A phalloidin potency assay was conducted for determining potential neutralization of N-001 by the ADA's described above. A known potent dose of N-001 (2.4 pmol) with a dilution series of the ADA containing sera from all replicate animals, was incubated with the cells. The potency assay was conducted with the combined ADA sera: N-001 mixtures and compared to N-001 treated and untreated cells (Fig. 5C). There was a normal amount of actin depolymerization from N-001 treated samples relative to the cells treated with N-001, and there was a similar amount of actin depolymerization for all the combined sera mixture dilutions for N-001 treated animals indicating that there is little to no loss in N-001 potency when combined with the ADA's. These results indicate neutralizing ADAs are not formed in response to repeated N-001 dosing.

## Discussion

In the present study, it was found that N-001 was effective against acute post-operative pain in the mouse hind paw incision model using both peri-incisional and popliteal nerve block drug application. The efficacy studies assessed the nociceptive (mechanical allodynia) and functional (gait) outcomes of N-001 in a mouse postsurgical pain mode^35^. Analgesia provided by N-001 significantly decreased mechanical sensitivity to evoked stimuli and improved gait after incision, while having a favorable profile in overall gait performance in uninjured control groups. The Basso Mouse Scale (BMS) was initially developed to assess functional locomotor recovery after spinal cord injury^36^. Along with other gait and weight bearing assessments of rodents, the BMS can be used as a surrogate for the measurement of nociception rodent models^37^. With the measurement of gait or weight bearing referred to as stimulus-independent nociception^37^, the combination of results from functional, stimulus-independent and stimulus-dependent nociceptive measurements result in a higher rate of translation to clinical settings^38,39^. Gait quality for incised animals was reduced compared to sham controls, while N-001 analgesia reverted treated animals back to normal gait. The result of N-001 restoring normal gait after paw incision provides supporting evidence for its effectiveness against nociception particularly when combined with stimulus-dependent efficacy results^24^. The increased duration of efficacy at 72 h was noticeably longer than current analgesics. Additionally the antinociceptive behavior in mice was reversible presumably because N-001 entered the cell by receptor-mediated endocytosis, limiting its intracellular amount where it reversibly modified actin^24^. The hind paw incision model used here for simulating post-operative pain has been recognized as a useful tool for drug testing and studying mechanisms of postoperative pain^40^. N-001 treatment of acute pain markedly ameliorated mechanical allodynia in mice and was found to be effective 2 h post-surgery while lasting at least three days. Additionally, the drug action was dose and time dependent, and mice injected at the MTD had no adverse side effects.

In the pharmacokinetic study, the elimination half-life of N-001 was relatively long (more than 72 h) after IP injection compared to commonly used analgesics such as Fentanyl, hydrocodone and morphine^41^. The PK profile was specific to the therapeutic component of N-001, C2I. Additionally, the non-linear C2I PK profile could be attributed to a non-uniform clearing mechanism of the drug. Target-mediated drug disposition (TMDD) is common for large molecules where the clearance is dependent on N-001 and its target^42^. The drug concentration in serum at the MTD had a multiple peak profile which may result from other forms of reabsorption into the serum promoting prolonged serum recirculation and result from enterogastric or hepatic reabsorption^43^. The drug at the MTD, was cleared from the system gradually after 48-h in both male and female mice. This is ideal for currently used treatment strategies to manage post-operative pain because it increases the duration of drug residency without elimination and may have improved efficacy by slowing drug clearance. Multiple injections of N-001 in mice did not trigger an adverse immune response over extended periods of time. The duration between doses was simulated to reflect the clearance of the drug and time taken for immune activation between each subsequent dose. In this case, emphasis was placed on detecting ADAs specific to the IgG isotype. It was also possible to detect both low binding and high binding isotypes through a detection mechanism specifically designed for this purpose where different titers of ADAs were used to check for neutralizing effects. Additionally, the titers obtained from mice injected with N-001 with increasing number of booster doses lacked neutralizing antibodies that would otherwise hinder the efficacy or compromise the safety of N-001.

In conclusion, the results indicate N-001 can provide relief from acute post operative pain and at a significant distance from the peripheral source of pain challenge. The absence of neutralizing antibodies even with repeated injections demonstrate the ability to use N-001 multiple times without loss of drug potency due to a neutralizing immune reaction. Efficacy demonstrated after paw incision and through a popliteal nerve block combined with past results using inflammatory and nociceptive pain models, further strengthen the efficacy of N-001 against a panel of nociceptive pain challenges. Since animals treated in this study returned to normal gait function and past results demonstrated in vivo and in vitro sensory neuron specificity, N-001 selectively acts on nociception without an effect on motor function^24,25^. Nociceptive pain relief apparent in the nerve block studies combined with previous results demonstrating targeting of specific neuronal subtypes^25^suggest that N-001 could be used as a differential nociceptor-specific nerve block. The TMDD clearance, longer efficacy compared to standard use analgesics, and absence of neutralizing antibodies, support the potential use of N-001 as a new first in class analgesic for post operative pain management. The combined current safety and efficacy data provide evidence that N-001 could be a nociceptor-specific nerve block agent without the side effects or safety concerns of previously studied drug candidates^44^.

## Materials and methods

### Reagents and antibodies

The N-001 was prepared as described previously^24,25^. In brief, plasmid encoded constructs for C2I and C2II-C1 were expressed in *E. coli* BL21. *E. coli* was cultured in LB medium supplemented with ampicillin (100 µg/mL) at 37 °C and were induced at an optical density of ~ 0.6 at 600 nm wavelength with 0.5 mM Isopropyl β-D-1-thiogalactopyranoside (IPTG). A French pressure cell was used to lyse the cell paste at 690 bar. Glutathione resin (Genscript) was used for affinity purification. GST fusion tags were removed using thrombin (Thermo Fisher). C2II-C1 was further activated using trypsin by incubation at 37 °C for 30 min at a 1:5 enzyme to substrate ratio as previously described^45^. A custom-made polyclonal antibody targeting the N-001 enzymatic component C2I, was prepared in rabbits. The other antibodies used were goat anti-mouse IgG (H/L) polyclonal antibody (BioRad, Cat no. STAR207P) and goat anti-rabbit IgG (H/L): HRP (BioRad, Cat no. STAR124P). Thermo Scientific Pierce TMB substrate was used for ELISAs.

### Animal care and use

Animal experimental procedures were performed in accordance with the National Institutes of Health Guide for Care and Use of Laboratory animals. All experimental protocols were reviewed and approved by Veterans Affairs Palo Alto Healthcare System Institutional Animal Care and Use Committee or the Institutional Animal Care & Use Committee of University of Nebraska-Lincoln depending on the site of the experiments and prior to beginning the work. Studies utilizing live animals followed recommendations of the ARRIVE guidelines. For the efficacy studies, male and female mice 10–11 weeks old of the C57Bl/6 J strain or BALB/c strain as indicated, were obtained from Jackson Laboratories (Bar Harbor, MA). Key behavior experiments were done in female efficacy experiments to ethically reduce the number of animals needed. For the safety experiments male and female mice 10–11 weeks old of the BALB/c strain were used from Jackson Laboratories (Bar Harbor, MA). All mice were kept in local animal facilities a minimum of 1 week prior to initiating experiments. The mice were housed in clean cages and kept under standard conditions with a 12 h light/dark cycle, an ambient temperature of 22 ± 1 °C and were allowed food and water ad libitum.

### Drug administration for efficacy studies

N-001 (3 pmol/10 μL), bupivacaine HCl (10 μL of 0.25%, 2.5 mg/mL) or carrier (10 μL) was administered locally by subcutaneous intraplantar (i.pl.) injections. Mice received either treatment, perioperatively or after hind paw incision was performed. Separate experiments evaluated the effects of the above treatments on incision after popliteal nerve block. The nerve block was done immediately prior to incision using the method described by Leszczynska and Kau^34^. N-001 (30 pmol/50 μL), bupivacaine HCl (50 μL of 0.25%, 2.5 mg/mL) or carrier (50 μL) was injected into the popliteal space.

### Hind paw incision

The hind paw incision model was performed as described previously^35,46^. Briefly, mice were anesthetized using isoflurane 2–3% delivered through a nose cone. After sterile preparation, a 5 mm longitudinal incision was made with a number 11 scalpel on the plantar surface of the right hind paw. This incision was sufficiently deep to divide deep tissues including the plantaris muscle longitudinally. After controlling bleeding, a single 6–0 nylon suture was placed through the midpoint of the wound and antibiotic ointment was applied. Testing took place at time points up to 7 days after incision.

### Nociceptive assay

Mechanical allodynia was assayed according to the “up-down” algorithm described by Chaplan et al.^47^. Previously we have applied this technique to detect sensitivity in mice after incision by using nylon von Frey filaments^35,46^. Mice were acclimated on the testing wire mesh platforms inside clear plastic enclosures (10 cm in D X 40 cm in H). Subsequently, sequential fibers with increasing stiffness were applied 1 mm lateral to the central wound edge and left in place 5 s. A response was defined as the withdrawal of hind paw from the fiber and then a less stiff fiber was applied; if no response was obtained the next stiffest fiber in the series was applied. Testing ended when 4 fibers had been applied after the first response was obtained.

### Functional gait analysis

To assess the effects of treatment on functional locomotor changes following incision, analysis of gait was done using the Basso Mouse Scale^48^. The Basso Mouse Scale is a 10-point locomotor rating scale (0 to 9). Animals with normal locomotion achieve a score of 9. The scale evaluates parameters including joint movement, stepping ability, coordination, and trunk stability. In addition, the more specifically gait-related Basso Mouse Scale subscore was also used. This subscore quantifies improvements in the areas of stepping frequency, coordination, paw position, trunk stability, and tail position.

### Toxicology

The toxicity of the drug was assessed following intraperitoneal (IP) injection of N-001 (0.8 pmol/g body weight in grams of the mice) or vehicle only (phosphate buffered saline) in 64 BALB/c mice (4 female, 4 male each at 4-time points). The animals were placed in cages with adequate nutrition and day/light cycle and assessed after 1, 3, 13 and 180 days. The animals were checked at regular time intervals for impairment, duress, and morbidity. The mice were euthanized at the respective time points and serum and whole blood was collected through terminal cardiac puncture. The samples were analyzed for Glucose, Creatinine, BUN, ALT, Total Bilirubin, Direct Bilirubin, Alkaline Phosphatase, LDH, AST, Indirect Bilirubin, BUN/Creatinine, Cholesterol, Triglyceride, Phosphorus, Calcium, Potassium, Sodium, Total Protein, Chloride, Albumin, Globulin, and Osmolality, and a Complete Blood Count (CBC test). The CBC test included white blood count, red blood count, hemoglobin, hematocrit, mcv (mean corpuscular volume, erythrocyte average size), mch (mean corpuscular hemoglobin), mchc (mean corpuscular hemoglobin concentration), rdw (red cell distribution width), platelet count, neutrophils %, neutrophils absolute, lymphocytes, lymphocytes absolute, monocytes %, monocytes abs, eosinophils %, eosinophils abs, basophils %, basophils abs.

### Pharmacokinetics

The pharmacokinetics of N-001 was evaluated following intraperitoneal (IP) injection of the MED (minimum effective dose) and MTD (maximum tolerated dose; 10 times MED) of N-001 8 pmol (MTD)/g body weight in grams of mice and 0.8 pmol (MED)/g body weight in grams of mice) or vehicle in BALB/c mice (4 female, 4 male each at 6-time points). The animals were placed in cages with adequate nutrition and day/light cycle and assessed after 2, 4, 8, 24, 48 and 168 h for mice injected with MTD and 2, 4, 8, 24, 72 and 96 h for mice injected with MED. The animals were checked at regular time intervals for impairment, duress, and morbidity. Blood was collected either through retroorbital bleeding or through terminal cardiac puncture. The serum was diluted in a serum diluent (MTD: 3:700, MED: 1:200) ratio before measuring the C2I concentration through ELISA. An indirect ELISA was designed where the antigen (tissue homogenates) in coating buffer (0.2 M carbonate-bicarbonate buffer; pH 9.74) was incubated in Nunc MaxiSorp™ high protein-binding capacity 96-well ELISA plates with binding capacity of 600–650 ng IgG/cm^2^ overnight at 4 °C. A custom made rabbit IgG anti-C2I was used as the primary antibody (1 μg/mL) whereas a goat anti-rabbit IgG HRP was used as the secondary reporter antibody (1:5000) which was used to oxidize the TMB-substrate and stopped with 0.2 M sulfuric acid, and absorbance at a wavelength of 450 nm was measured.

### ADME studies

The ADME of N-001 was evaluated following intraperitoneal (IP) injection of the MED of N-001 (0.8 pmol/g body weight in grams of mice) or vehicle only (phosphate buffered saline) into 64 BALB/c mice (4 female, 4 male each at 4 times). The animals were placed in cages with adequate nutrition and day/light cycle and assessed after 1, 3, 13 and 180 days. The animals were checked at regular time intervals for impairment, duress, and morbidity. The mice were euthanized at the respective time points and tissues (skin, brain, liver, kidneys, spleen, pancreas, reproductive organs, heart, lungs, colon, small intestine, and stomach) were harvested. The tissue homogenates were prepared by cryofreezing tissue and use of a mechanical homogenizer in a RIPA buffer with protease inhibitor^49^. The homogenates were characterized for total protein concentration using a BCA assay. The homogenates were now used with an ELISA in 1:200 and 1:100 dilution in blocking solution (1% Bovine serum albumin in PBS; pH 7.2) to identify the concentration of C2I present in them. An indirect ELISA was designed where the antigen (tissue homogenates) in coating buffer (0.2 M carbonate-bicarbonate buffer; pH 9.74) was incubated in Nunc MaxiSorp™ high protein-binding capacity 96 well ELISA plates with binding capacity of 600–650 ng IgG/cm^2^ overnight at 4 °C. A custom made rabbit IgG anti-C2I was used as the primary antibody (1 μg/mL) whereas a goat anti-rabbit IgG HRP was used as the secondary reporter antibody (1:5000). Absorbance produced by HRP mediated reduction of hydrogen peroxide and oxidation of TMB-substrate was measured after stopping the reaction.

### Titration antibody assay

Animals (Females, Controls n = 6; Experimental test samples (N-001) n = 12) were injected with (0.8 pmol/g body weight in grams of the mice) of N-001 through IP route of administration on day 1 and a booster dose of the drug three more times every 7 days. The IP route of administration was chosen because of the flexibility in dosing and easier distribution profiles compared to subcutaneous dosing method. Blood was harvested from mice through retroorbital bleeding 5 days after IP injection and an additional 10 days after the final booster injection. The experimental design was used to evaluate the formation of anti-drug antibodies produced in the mice with repeated dosage and possible neutralizing antibodies produced against the therapeutic component of N-001. The design of experiments was intended to mimic the use of N-001 as a post-surgical analgesic. An indirect ELISA was designed where the antigen (N-001; 100 ng/mL) in coating buffer (0.2 M carbonate-bicarbonate buffer; pH 9.74) was incubated in Nunc MaxiSorp™ high protein-binding capacity 96 well ELISA plates with binding capacity of 600–650 ng IgG/cm^2^ overnight at 4 °C. Serum samples were diluted initially to 1:100 ratio in General Serum Diluent (Immunochemistry Technologies, LLC) with 0.01% sodium azide followed by further fourfold titrations (1:16; 1:64; 1:256; 1:1024; 1:4048) which were incubated with pre-coated plates with N-001. A goat anti-mouse IgG HRP was used as the secondary reporter antibody (1:5000) which was used to oxidize the TMB-substrate and the reaction was terminated using 0.2 M sulfuric acid, and the subsequent absorbance at a wavelength of 450 nm was measured. The presence of antidrug antibodies (ADAs) was directly proportional to the intensity of the absorbance measured. Controls were used to compare the background including non-specific interactions caused due to the serum. The antidrug antibodies were analyzed for the C2I (enzymatic) region of N-001 drug.

### Neutralizing assay

The presence of neutralizing anti-drug antibodies (ADAs) were determined using a previously described potency assay^24^. Serum collected from BALB/c mice that received 4 separate doses of N-001 at 0.8 pmol (MED)/g body weight in grams of the mouse injected every 7 days. Neuroblastoma cells (SH-SY5Y) were used to test neutralization of ADA containing serum using a previously described method of quantitative staining^24^. The SH-SY5Y cells were plated onto black sided 96 well plates (Corning) at a cell density of 120,000 cells/mL. Cells were allowed to incubate at 37 °C for 48 h in culture media. Following incubation, cells were treated for 2 h with N-001 at 2.4 pmol or 2.4 pmol N-001 mixed with ADA containing serum from 21 days after the final N-001 injection at the following dilutions: 1:125, 1:256, 1:1024, 1:4048 for n = 12 (biological replicates) and N = 3 (technical replicates). After incubation, the cell culture medium was removed and washed with PBS. Cells were fixed with 4% paraformaldehyde for 15 min at room temperature. Cells were washed 3 times with PBS then permeabilized with 0.5% (v/v) Triton X-100 for 10 min, then washed with PBS 3 times. Cells were stained with Alexa Fluor™ 488 Phalloidin (Invitrogen, Cat no. A12379) for 30 min at room temperature. Fluorescence was quantitated using a plate reader at Excitation _485 nm_/Emission _525 nm_.

### Statistics

Statistical significance for all efficacy data was determined by a two-way ANOVA test, followed by a Sidak post-hok test. Significance was displayed as *p *< 0.001 (***), *p *< 0.01 (**), and *p *< 0.05 (*).

## Supplementary Information

Supplementary Figures.

Supplementary Information 2.

Supplementary Information 3.

## Data Availability

The supplemental data section includes raw datasets from safety studies performed in this study. Remaining datasets used and/or analyzed during the current study available from the corresponding author on reasonable request.
