# Supplementary material for: Preclinical characterization of the efficacy and safety of biologic N-001 as a novel pain analgesic for post-operative acute pain treatment
Source: Sci Rep. 2023 Jul 21;13:11778. doi: 10.1038/s41598-023-38618-4 (PMC10362049; doi:10.1038/s41598-023-38618-4)
Supplement: Supplementary file 1 — Supplementary Figures. [file 41598_2023_38618_MOESM1_ESM.pdf]

A

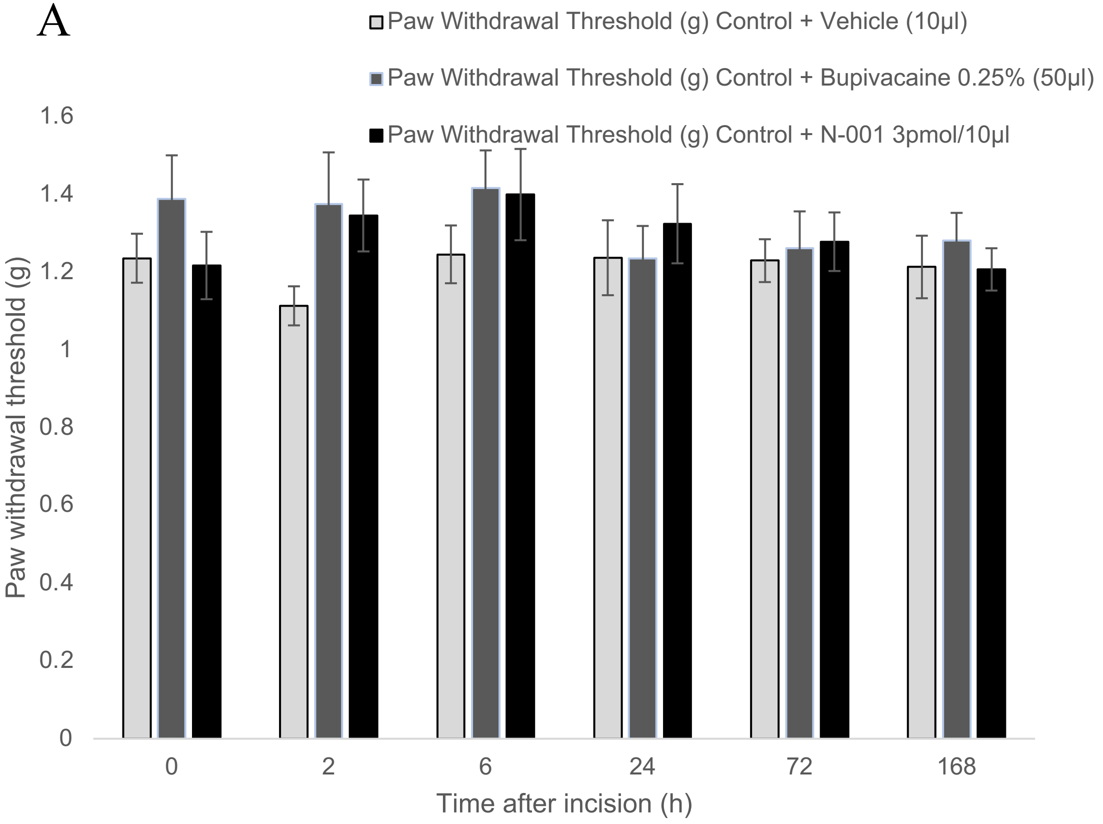

B

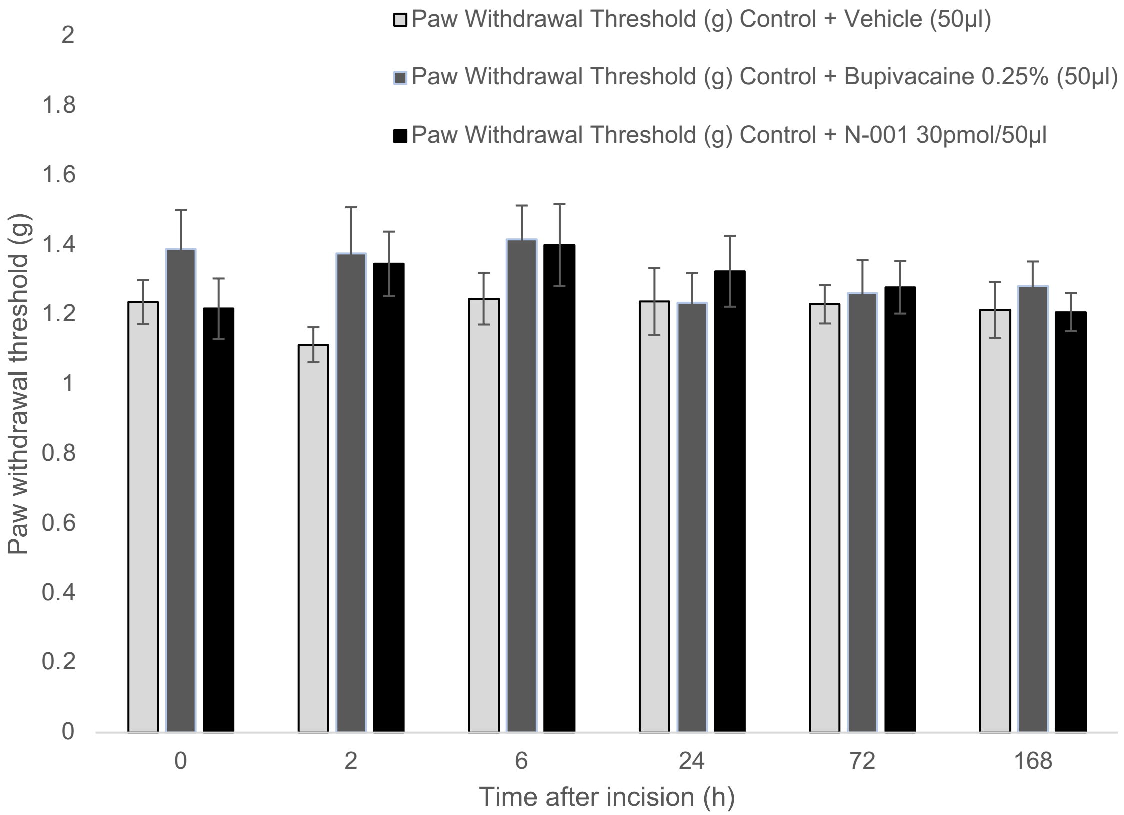

Figure S1. **Figure S1A.** Hindpaw withdrawal threshold of C57Bl/6J male mice with control and vehicle/drug treatment, n=8. **Figure S1B.** Hindpaw withdrawal threshold of C57BI/6J male mice after control procedure and vehicle/drug (50 µl Popliteal Block) treatment, n=8

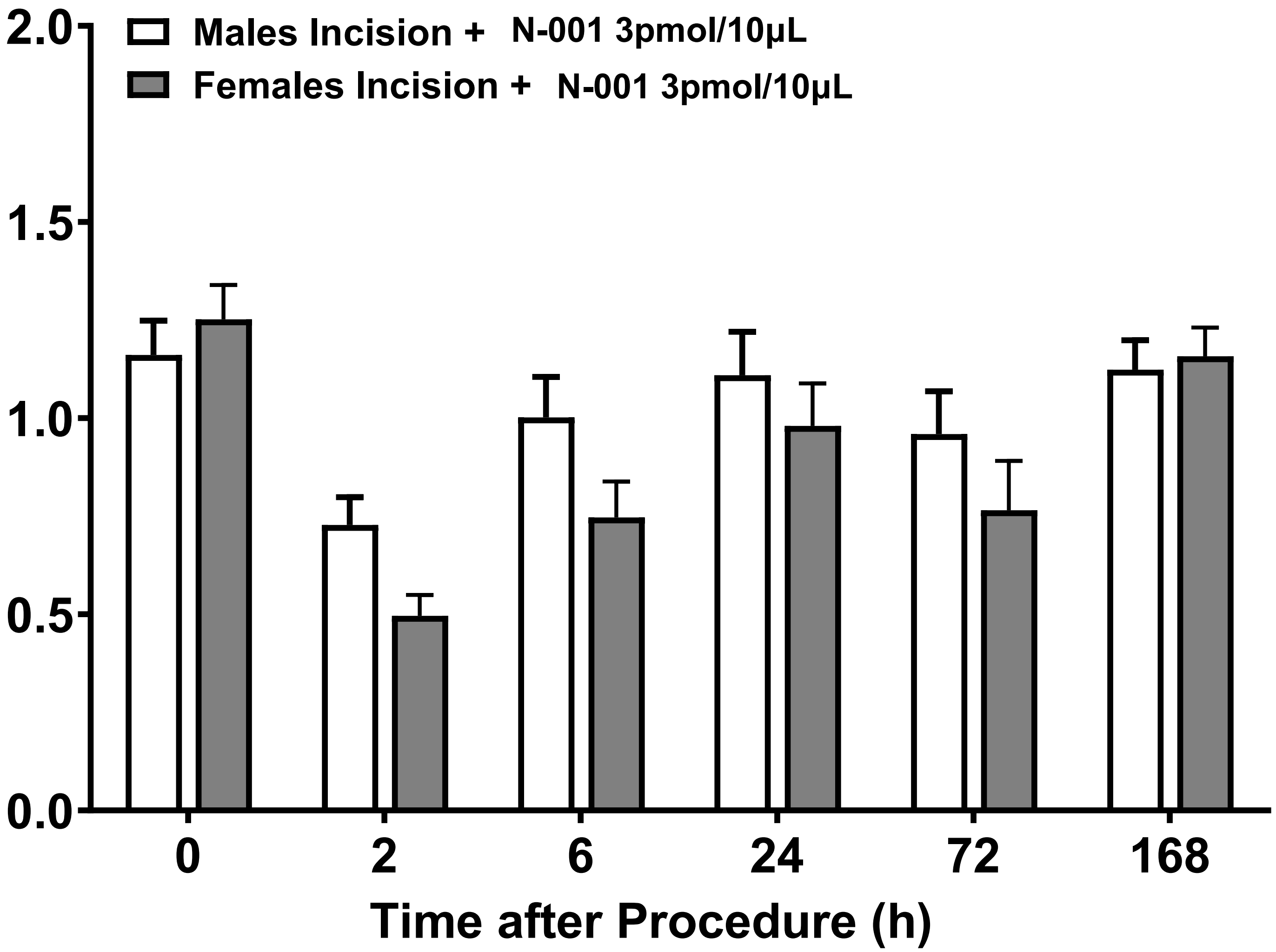

***Figure S2*.** Hindpaw withdrawal threshold of C57Bl/6J male vs female mice after incision and vehicle/drug treatment.

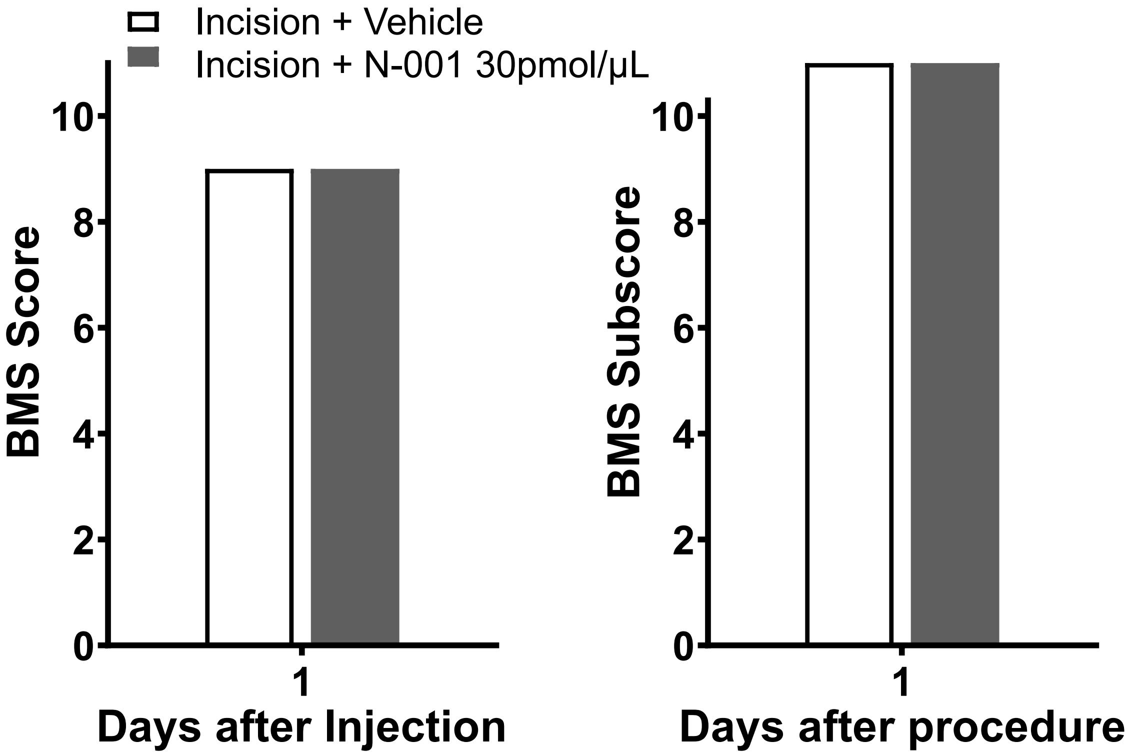

Figure S3. Basso Motor Scale (BMS) of C57Bl/6J male mice before and after incision and vehicle/drug control procedure and vehicle/drug (50 μl Popliteal Block) treatment, n=8

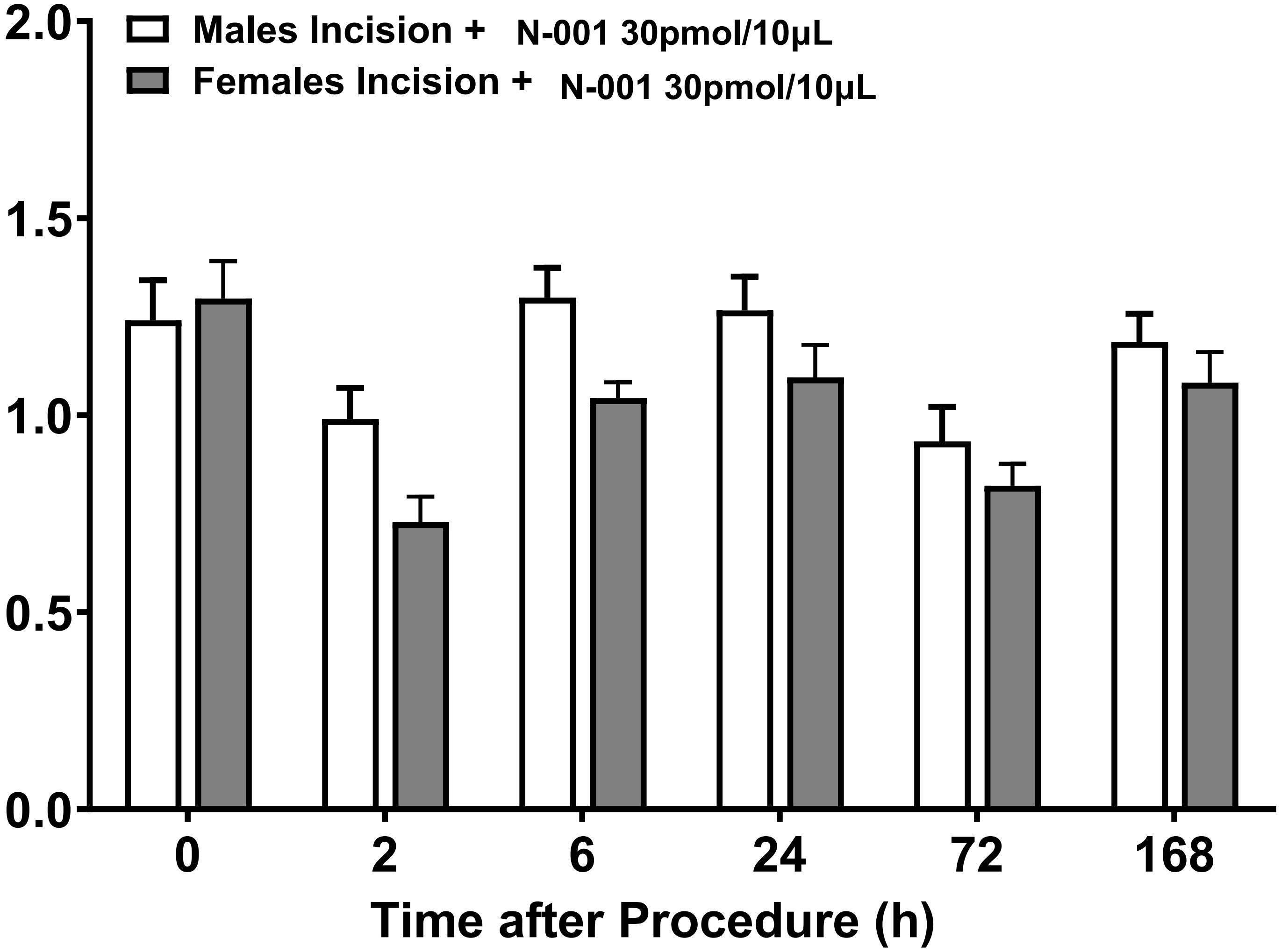

Figure S4. Hindpaw withdrawal threshold of C57Bl/6J male vs female mice after incision and popitieal nerve block
.
